# Literacy as part of professional knowing in a Swedish dental education

**DOI:** 10.1186/s12909-021-02800-x

**Published:** 2021-07-08

**Authors:** Viveca Lindberg, Sofia Louca Jounger, Maria Christidis, Nikolaos Christidis

**Affiliations:** 1 Department of Humanities and Social Studies Education, Stockholm University, SE-106 91 Stockholm, Sweden; 2 Division of Oral Diagnostics and Rehabilitation, Department of Dental Medicine, Karolinska Institutet and Scandinavian Center for Orofacial Neurosciences (SCON), SE-14104 Huddinge, Sweden; 3 The Swedish Red Cross University College, The Institute of Health Sciences, SE-141 21 Huddinge, Sweden

**Keywords:** Dental education, Professional literacy, Academic literacy, Professional content, Teaching/learning

## Abstract

**Background:**

Academic reading and writing are seen as self-evident literacy competences in most contemporary higher educations, however, whether students also are introduced to professional literacy of relevance for dentistry during their education is a question. The purpose of this study is to analyze one of the Swedish dental programmes, with respect to its design, in relation to possible content of relevance for academic and professional literacy. Secondarily, to identify and analyze Swedish dental students’ writing in an academic setting, i.e. what these students are expected to read and write, and how they write.

**Methods:**

Data, for this ethnographically inspired case-study, was produced by observations and audio-recordings of lectures, copies of teachers’ handouts and of volunteering students’ notes, and a multiple-choice-test. Data-analysis was made in five steps, starting with macro-level data, i.e. curriculum and syllabuses, followed by the syllabuses for the two observed modules, the teacher-provided material, analysis of the students’ notes, while in the fifth and final step, the results from the previous steps were compared, to find patterns of *what* students were expected to read and write, and *what* in the teacher-provided multimodal material that was emphasized in teachers’ talk.

**Results:**

This study showed that students were engaged in several types of literacy events, such as *reading*, *finding and watching videos* on their learning platform, *writing*, and *following instructions*. The study also showed that there is a recurrent academic content comprised of anatomy, physiology and pathology, while the professional content comprised of patient communication and anamnesis. Further, an integrated content was found and was initiated in teacher-constructed PowerPoints and by student-questions. Note-taking patterns varied between individual students, but the general pattern for this group of students were the use of complementary notes. This type of note-taking was used to make available further descriptions of the teacher-constructed text in PowerPoints, but also an independent text describing pictures shown on teachers’ PowerPoints or the blackboard.

**Conclusion:**

Findings from the present study reveal that students either copy text from teachers’ PowerPoint-slides, re-formulate text from teachers’ PowerPoint-slides, or write complementing text to teachers’ PowerPoint-slides. Further, the students individually choses type of note-taking based on situation. The study also revealed that the academic literacy – in the two modules during the fifth and sixth semesters of a dental education analyzed – mainly has a professional basis for reading, writing, and communication purposes. The study also showed that academic and professional literacy are closely connected through recurrent integration.

## Background

Dental education is one of several professional programmes in Swedish higher education. The national learning outcomes stated for degree in dentistry in the Higher Education Ordinance (SFS 1993:100) [1] emphasize the importance of scientifically based knowledge as well as of proven experience for dental work. This includes not just the capability of making diagnoses, treatment plans, as well as treating various dental diseases and malformations, but also leadership and collaboration. Such formulations of skills are abstract, as they are embedded in different learning outcomes [2] across the entire program. The learning outcomes are then specified in each course syllabus. Together these courses then form the educational programme. In most professional programmes within higher education parts of the learning outcomes relate to the *theoretical* aspects of dental work and parts that relate to the *practical* (clinical) aspects of dental knowledge. The *theoretical* aspects are for instance content like anatomy and physiology, or content that relate to tools, materials and their properties, that are used for dental work. The *practical* (clinical) aspects of dental knowledge relater to what dentists actually do. Aspects of these are both understanding patient-needs, hands-on skills, being able to take an anamnesis and transform it to the patient record, as well as being able to explain oral conditions, treatment need and prognosis [3]. Academic reading and writing are seen as self-evident literacy competences in most contemporary higher educations. However, what and how you write is not simply a matter of being able to read and write. Contemporary research shows that even academic literacies are plural and situated in context: academic programmes differ in what kinds of text they read but also in how they read these texts, that is, the purpose of reading matters in relation to how you read [4–6]. Likewise, analyses of texts written within different disciplines has shown that patterns in how you write differ between academic contexts. Consequently, successful students learn how to master the *literacy practices* of a program, that is, what kinds of texts they are expected to read and write, what qualifies as acceptable texts within the program, and alter between types of texts for the different purposes that occur within their program. These kinds of studies occur within anthropology, linguistic studies and educational studies or in cross-disciplinary studies with interest in situated literacies. This article is based on a common interest in the literacy practices part of dental education. But academic programmes that prepare for specific professions also need to consider the literacy practices of the specific profession. While some researchers have specialized on academic literacies, others have specialized in vocational literacies [7–9]. Although it is obvious that it takes time to become a skilled dentist, it seems less obvious that it also takes time to become a skilled writer in academia. Hence, dental work also requires professional literacy competence.

This study is framed within the tradition of New Literacy Studies (NLS) [4, 10], which means focusing on literacies as social practices [5] rather than the acquisition of basic skills in reading and writing. Such an approach recognizes multiple literacies. In other words, the meaning of being a skilled reader or writer varies between contexts. A common way of describing a literacy practice is with the help of answering the following (analytical) questions: *what* are participants in a specific practice expected to read?; *How* should these texts be read?; and *For what purposes* are they read? Similar questions are posed to writing: *What* are participants in a specific practice expected to write?; *How* are such texts expected to be written?; and For *what purposes* are the texts written? [11–14]. Later studies [15] emphasize the need of studies that also address communicative activities related to texts: “where written texts meet spoken conversation” (p. 1), that is how people talk about present or absent texts [16]. Many NLS studies in higher education have focused the characteristics of literacy practices in academia, i.e. what literacy competencies and strategies are needed for educational activities within different disciplines [5, 11].

Characteristic for academic literacies, whether in science [14], in history, or in national economy [12], is that students need to master a breadth of literacy practices [17]. Further, the students also need to master progression, in terms of complexity, in order to become successful within a programme or a discipline [18–20]. Being a student in higher education assumes participating in activities that to a large extent are literacy-based. To have or to develop literacy competencies and strategies needed for educational activities are crucial resources for students. These are the means for coping with literacy demands in varying but relevant situations, depending on the purpose of reading and or writing.

The transition from upper secondary education to academia is sometimes hard, especially when the literacy practices differ from the ones students are familiar with [21]. However, professional programmes in higher education not only prepare for literacy practices within academia, but they also prepare for professional literacy practices, relevant for each profession. Transition from higher education to work requires that students can cope with the literacy practices of the profession. Studies with this focus show that although a person may be a successful student, (s) he is not necessarily well acquainted with the kinds of literacy demands that (s) he will encounter when entering the world of work [7, 8, 22], and, of course, the other way around.

When it comes to literacy within dental education, we found a few studies of relevance for our study. One study, concerned the use of digital photographs in clinical work of a dentist as well as in communication with other professionals [23]. Other studies discuss either digital literacy at an early stage in dental education [24], or students’ conceptions of digital tools in dental education [25], or the matter of collaboration between students in other health education programs as well as licensed dentists [26]. A conclusion from the studies within the field is that, although studies of literacy in higher education already has been focused for two decades, literacy in dental education has not yet been addressed. Thus, NLS serves as a productive theoretical framework for studying literacy in a dental context, and this study complements the few existing studies within dental education.

Dental education in Sweden is based on a *curricular system* [27] consisting of various documents: from national laws, ordinances and admission requirements to local curricula, examinations and assessment [28], but also demands related to the European agreement based on the Bologna Process, for a harmonization of higher education to a common system, the European Higher Education Area [29, 30]. For this study, the documents in the curricular system in focus are the Study Programme in Dentistry (SPD) at Karolinska Institutet (KI), specifically the curricula for the compulsory courses of this programme, as well as the syllabuses for two modules in the programme. Our interest is directed to classroom practices (lectures, clinical training), and we focus on the contexts in which the various literacy events occur.

The purpose of this study is to analyze one of the Swedish dental programmes, with respect to its design, in relation to possible content of relevance for academic and professional literacy. Secondarily, to identify and analyze Swedish dental students’ writing in an academic setting, i.e. what these students are expected to read and write, and what they write.

## Methods

### Context of the study

The SPD has a duration of 5 years, equal to 300 European Credit Transfer and Accumulation System (ECTS), divided into 10 semesters. After graduation the newly examined students have to apply for a licensure from The National Board of Health and Welfare (https://legitimation.socialstyrelsen.se/sv/utbildad-i-sverige/tandlakare) in order to be allowed to work as a dentist. The total number of full-time students at the SPD (when the study took place) was 423, divided into approximately 85 students per academic year.

The first 3 years of the SPD are on a basic level, while the last two on an advanced level. The students are supposed to develop a clinical maturity, empathy and mature in their role as health-care providers. During the following 2 years the students are deepening their knowledge upon diagnosis and treatment, to learn about rules and regulations, quality assessments, scientific evaluations and reflect upon present evidence, start treating more advanced patient cases such as children, elderly and patients with orofacial pain and jaw dysfunction (https://ki.se/selma/programme-syllabus/2TL13). Each of the 10 semesters is composed by courses and many courses divided in modules (https://pingpong.ki.se/public/courseId/6128/lang-sv/publicPage.do?item=3886077). The third year, which is the focus of this paper, consist of the fifth and sixth semesters. The modules “Orofacial Pain and Jaw Function 1 and 2” (OPJ) are closely connected with each other and are separated by the semester break. During the fifth semester the module is part of the course called Clinical odontology 2 (21 credits), while it during the sixth semester is part of the course Clinical odontology 3 (24 credits).

An ethnographical inspired approach was used to study literacy practices in the students’ academic environment [31–33]. Although the collection of research material was conducted during recurrent shorter time-periods, based on the structure of the SPD, the study can still be considered as ethnographically inspired since various kinds of data was collected over a period of time [31, 34, 35], i.e. from November 2017 to February 2018, during the third year of the SPD, during the modules OPJ 1 and 2, at the Department of Dental Medicine, Karolinska Institutet, Huddinge, Sweden.

### Data collected

For this case study the following data was collected: observations and audio-recordings of lectures, copies of teachers’ handouts and copies of students’ notes (from those who volunteered) (Table 1), multiple-choice test. Since the focus is on literacy practices, ethnographic mapping of literacy events during lectures is significant [12, 33, 36–38]. A literacy event is defined as “activities where literacy has a role. Usually there is a written text, or texts, central to the activity and there may be talk around the text. Events are observable episodes which arise from practices and are shaped by them” [39]. In the following, these are described closer.

**Table 1:** Quantitative descriptions of student texts

Student	Number of pages	Handwritten notes	Digital notes
1	7 pages	7 pages	0 pages
2	38 pages - 4 slides per page	0 pages	38 pages - on PowerPoint-copy
3	16 pages	6 pages	9 pages
4	10 pages	7 pages	3 pages
5	1 page	0 pages	1 page
6	13 pages - 4 slides per page	0 pages	13 pages - on PowerPoint-copy
7	21 pages - 4 slides per page	0 pages	21 pages - on PowerPoint-copy
8	20 pages - 4 slides per page	0 pages	20 pages - on PowerPoint-copy
9	1 page	0 pages	1 page
10	3.5 pages	0 pages	3.5 pages
	∑ 129.5 pages	∑ 20 pages	∑ 109.5 pages

### Participants - teachers and students

Both lecturing teachers accepted participation and handed in the handouts from the lectures. They are registered specialists in orofacial pain and jaw function with long clinical experience of more than 10 years, and long teaching experience for more than 15 years. One was a female professor (hereby referred to as FP) and 1 a male associate professor (MAP).

In total, 68 students attended the modules. Of these, 49 were women and 19 men. Their ages varied from 20 to 42 with an average of 24.8 and a standard deviation of 4.4. Ten students handed over their anonymized notes from the lectures, and three students, a male (M1) and two females (F1; F2), reported interest for participation in the interviews. Results from the interviews are presented in another article by Lindberg and co-workers from 2020 [40].

### Module

The first module, during the fifth semester, comprised of five lectures of 45 min duration each including the following content: **a)** Introduction to orofacial pain; **b)** The temporomandibular joint; **c)** The masticatory muscles; **d)** Occlusion; and **e)** Temporomandibular disorders (TMD). Complementary to the lectures there was one clinical session of 4 h duration including alginate impressions of the jaws as well as bite-registration, for ordering an occlusal appliance.

The second module, during the sixth semester, comprised of three lectures of 45 min duration each including the following content: **a)** Clinical examination of the temporomandibular region; **b)** Treatment of TMD with occlusal appliances; and **c)** Other treatment modalities of TMD. Complementary to the lectures there were five clinical sessions of 4 h duration each including: **1)** Clinical examination of the temporomandibular region including analysis of the occlusion; **2–4)** Adjustment and delivery of occlusal appliances; **5**) Follow-up examination, adjustment of occlusal appliance as well as a digital multiple-choice examination.

The material collected from the clinical sessions in OPJ 1 and 2 will be analyzed and presented elsewhere [40].

### Teacher provided material

The teachers sent by e-mail, all handouts (in total seven, one for each lecture) consisting of 267 slides. They also provided clinical instructions including one Excel-sheet with suggestions of phrases for patient notes as well as eight Word-pages of clinical instructions for examination and control of occlusal appliances. They also provided the curriculum for the SPD as well as the syllabus for the module OPJ. The module literature, recommended however not compulsory, was *Management of temporomandibular disorders and occlusion* by Jeffrey P Okeson (Ed.). The book is on 488 pages (of which approx. 100 pages consist of references). Of the 68 students, 16 bought the book.

Other teacher provided material were the multiple-choice test and students’ results. For the clinical skills demonstration, no documentation is demanded of the students. The teacher fills in a template for each student based on observation of students’ performances. The skills demonstrations are assessed with pass or fail. These are though not included in this article [40].

### Student provided material

Out of 68 students, 10 students handed in anonymized notes from the eight lectures, presented in Table 1. These notes from were classified in relation to whether they were handwritten or digital (Table 1).

### Participant observation

In line with studies framed by NLS, we also complemented the written material provided by teachers and students with ethnographic mapping [12, 33, 36, 38] All lectures were audio-recorded (in total 10 h 36 min and 11 s), with the microphone placed by the teacher in question. One or two of the researchers attended each lecture and were present as observers, taking field notes of *literacy events*, that is, teachers’ lectures (communication *to* students) and possible communication *with* students in relation to their PowerPoints, what students read or wrote, and their text-related communication (questions *to* teachers). For each slide in the PowerPoints we noted how much time was spent on teachers lecturing, questions or comments from students of relevance for the module.

### Analysis

Analysis of the data was made in five steps, starting with macro-level data (curriculum and syllabuses) in order to identify where in programme academic and professional literacy are indicated in the objectives [37], that is, the overall literacy indicated in the SDP (the intended curriculum [41, 42];. These are firstly summarized in Table 2, and secondly categorized based on the following analytical questions [31, 43]: *what* are students expected to read and write and *for what* purposes?

**Table 2:** Key findings based on the questions and definitions in the modules “Orofacial pain and jaw function 1 and 2”

**Discipline**	**Field of study, academic subject**
	**Field of study:** the Study Programme in Dentistry
	**Academic subject:** Orofacial Pain and Jaw Function
**Question**	**How do genres/modes vary across the two modules?**
	**Teachers:** *from* PowerPoints based on course literature, including teachers´ clinical experiences, *to* clinical instructions
	**Students:** *from* academic notes *to* clinical literacy
**Genre**	**What types of text occurs across the modules?**
	**Teachers**: PowerPoints based on course literature, complemented with teachers’ clinical experiences, and clinical instructions.
	**Students**: notes
**Genre modes**	**What regularized, organized sets of resources for meaning-making are made available for students?**
	**Speech:** examples of teachers’ clinical experiences.
	**Handout copies of slides:** anatomical, physiological, and pathological illustrations, photographs as well as video-clips.
	**Clinical instructions:** written instructions step-by-step including illustrations and photographs, demonstrations including speech, gaze, gestures and hands-on movements.
	**Digital resources:** online-accessible web-platform (CANVAS) with information regarding course-information, e-examination as well as handout copies of slides and clinical instructions.
**Switching/ transformation**	**Changing meanings and representations from one mode (e.g. speech) into another mode (e.g. writing), often involving a different mix of both modes (e.g. writing and layout)**
	**Teachers**: Exemplifying text using white-board, gestures, teachers’ sharing of clinical experiences
	**Students**: Questions to text, to teachers´ clinical experiences, to clinical instructions

In a second step we made a close analysis of the syllabuses for the two modules of OPJ that were in focus of our study. Here we included not only the objectives but also the content of the syllabuses for identifying what academic and professional literacy was expected from the students. In this step, new actors add their interpretations of the education plan [44, 45]. Complementary to the analytical questions above, we added a third question: *what* (if any) indications are there of *how* students are expected to read and write?

In the third step, we analyzed the teacher provided material (PowerPoint-slides). In general, the slides varied in terms of amount of text, plain text or text in combination with pictures. We classified the slides in four categories: text only, text with decorative pictures, informative pictures complemented with short informative text (terminology) or a combination of informative pictures and full sentences. Slides in each category where then counted in order to establish the proportions between the different types of slides (Table 3). Furthermore, we checked our notes from the lectures, in order to find for how long the teachers talked in relation to the different kinds of slides (Table 3). So far, our focus was on the quantitative aspects of slides as well as teachers’ talk. During lectures, 1–2 observers took field-notes, marking the time the respective teachers talked in relation to each PowerPoint-slide. The total number of slides for the seven lectures was *N* = 267, distributed on each lecture as follows: lecture 1 (*N* = 34); 2 (*N* = 28); 3 (*N* = 40); 4 (*N* = 39); 5 (*N* = 44); 6 (*N* = 53); and 7 (*N* = 29). When this was done, we listened to the recordings to find qualitative differences between teachers’ talk within a certain category of slides as well as between the different categories of slides. For the slides where the communication related to the slides was longer, we posed analytical question like: *What slides are paid more attention in terms of either teacher spending more time on them or students asking questions about them? What characterizes the content in these slides? What is emphasized in these? What (if any) are students’ questions to these slides?* Hence, the analytic questions for the teacher provided material can be summarized to (Table 2): *what* do they talk about? *How* is talk related to text? *What* do they emphasize? *What* (if anything) seems to be recurring issue?

**Table 3:** Courses in the Study Programme in Dentistry with objectives related to academic or professional literacy

Semester	Focus on profession	Focus on academia
**1st semester**		
a. To become a dentist 1, *7,5 credits* b. Scientific introduction, *3 credits*	Communication between patients and care-givers *(a)*^a^ Practical training in writing patient record *(a)* Differences between private and professional conversations *(a)*	Scientific articles and their structures *(b)* Introduction to library *(b)*
**2nd semester**		
a. To become a dentist 1, *7,5 credits*	Communication between patients and care-givers *(a)* Practical training in writing patient record *(a)* Differences between private and professional conversations (continues) *(a)*	
**3rd semester**		
c. Clinical Odontology 1, *24,5 credits*	Documentation (reading and writing patient records) *(c)*	
**4th semester**		
c. Clinical Odontology 1, *24,5 credits* d. To Become a Dentist 2, *1,5 credits*	Documentation (reading and writing patient records) *(c)* Ethical rules and considerations *(d)*	Ethical rules and considerations *(d)*
**5th semester**		
e. To become a dentist - Teamwork, *1,5 credits*	Communication in teams (dentist with nurse, dentist with dental hygienist, dentist with dental technician) *(e)*	
**6th semester**		
f. To become a dentist 3, *3 credits* g. Degree Project in Odontology, *30 credits*	Communication psychology *(f)* Motivational interviewing *(f)*	Introduction to thesis-writing *(g)* Ethical rules and considerations *(g)* Discussion of study design and methods *(g)*
**7th semester**		
g. Degree Project in Odontology, *30 credits* h. Children’s and adolescents’ dentistry, *22,5 credits*	To communicate with children and adolescents *(h)*	Academic writing: Ethical rules and considerations *(g)* Discussion of study design and methods *(g)* Database searches including strategies, critical review *(g)*
**8th semester**		
g. Degree Project in Odontology, *30 credits* h. Children’s and adolescents’ dentistry, *22,5 credits* i. Clinical Odontology 5, *10,5 credits*	To communicate with children and adolescents in the clinical setting *(h)* To communicate with children and adolescents in a community setting *(h)* Patient history (anamnesis taking and writing) *(h, i)*	Academic writing: Ethical rules and considerations *(g)* Discussion of study design and methods *(g)* Database searches including strategies, critical review *(g)* Half-time presentation
**9th semester**		
g. Degree Project in Odontology, *30 credits* j. Clinical Odontology 6, *11,5 credits* k. Oral Surgery, *4,5 credits* l. Geriatric Dentistry, *4,5 credits*	Patient history (anamnesis taking and writing) with young adults, adults, and elderly patients *(j, k, l)*	Academic writing: Ethical rules and considerations *(g)* Discussion of study design and methods *(g)* Database searches including strategies, critical review *(g)* Writing of degree thesis in odontology *(g)*
**10th semester**		
g. Degree Project in Odontology, *30 credits* k. Oral Surgery, *4,5 credits* l. Geriatric Dentistry, *4,5 credits* m. To Become a Dentist 5, *3 credits* n. Clinical Odontology 7, *12,5 credits*	Patient history (anamnesis taking and writing) with young adults, adults, and elderly patients *(k, l)* Administration and regulations within the field of odontology *(m, n)* Strategies for life-long learning and career-planning *(m)* To analyze and judge performed treatment, as well as need for referral *(n)* To set revision-plans *(n)*	Academic writing: Ethical rules and considerations *(g)* Discussion of study design and methods *(g)* Database searches including strategies, critical review *(g)* Writing of degree thesis in odontology *(g)* Presenting degree thesis in odontology *(g)* Oral and written critical review of peer’s degree thesis in odontology *(g)*

^a^ Letters in brackets refer to courses listed in the column Semester

**Fig. 1:**
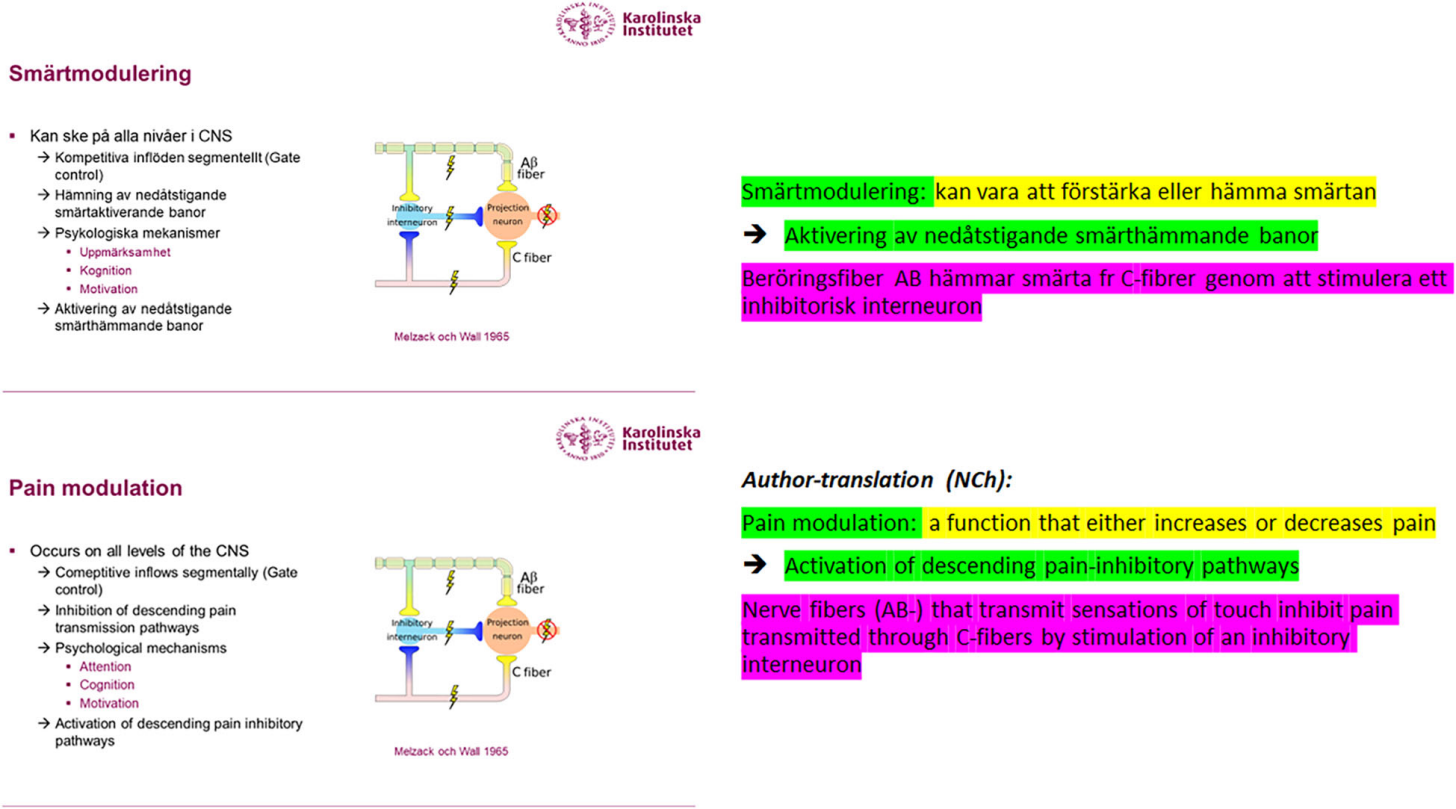
Example of color coding of students’ notes. Green color was used for marking copied text, yellow color was used for marking reformulated text, and purple color/orange color was used for marking complementary text

In the fourth step we analyzed students notes. They were firstly compared to teachers’ handouts (copies of PowerPoints) to students, and analyzed in relation the question what do student write in their notes? Color-coding was used for this procedure. Green color was used for marking copied text, yellow color was used for marking reformulated text, and purple color/orange color was used for marking complementary text, as exemplified in Fig. 1.

This analysis was performed by two researchers, one specialized in dentistry (SLJ) and the other in pedagogy MCh), for reasons of double control. Hence, every student’s notes were read by two researchers simultaneously and independently and compared to the information in the teacher-constructed PowerPoints. Both researchers had participated in data collection and observation of lectures. The analysis was developed in two steps, a control analysis comprising randomly selected notes from one student, and a main analysis of all student texts. Control analysis was then compared to main analysis. In cases where judgements differed, the two researchers shared their arguments for their judgements and agreed on which argument had best support in order to achieve a satisfactory congruence. Examples of differences in judgement were found to be related to core-content knowledge. Secondly, the amount of color markings was then estimated, on a quantitative basis, and patterns of the three types of notes (copied, re-formulated, or complementary) for each student were identified. *Copied notes* indicate that students only copied text from teachers’ PowerPoints; *re-formulated notes* indicate that students re-wrote the text from teachers’ PowerPoints using their own words; while *complementary notes* indicate that students added information such as teacher-provided examples, illustrations and explanations to the content in the PowerPoints. The types of patterns possible to discern based on these data were firstly an exclusive use of one type of notes, a dominating use of one type of notes or a mixed type of notes. Thirdly, for students who showed a dominating type of note, we looked for whether a pattern for complementary notes could be discerned. These patterns were then constructed into profiles for students’ notetaking (Table 4).

**Table 4:** Profiles for students’ types of notes

Student	Exclusively	Dominating	Mixed
1		Complementary text *In combination with copied and re-formulated text*	
2	s	Complementary text *In combination with re-formulated text*	
3		- Digital notes: complementary text *In combination with re-formulated text* - Handwritten notes: copied text *In combination with re-formulated text*	
4			Copied & complementary text *Equally distributed*
5		Complementary text *In combination with re-formulated text*	
6		Complementary text *In combination with re-formulated text*	
7		Complementary text *In combination with re-formulated text*	
8		Complementary text *In combination with re-formulated text*	
9			Copied & complementary text *Equally distributed*
10			Copied text, re-formulated text and complementary text *Equally distributed*

In the fifth and final step, we compared the results from the previous steps in order to find patterns of what students were expected to read and write and what in the teacher-provided multimodal material that was emphasized in teachers’ talk. For this analysis, we used the questions and definitions suggested as shown in Table 2, based on the study by Lea and Street (2006) [46], modified to the present setting. In combination, these questions generate answers regarding the lecturing practice at KI, the content of the course and its possible variation in importance in a specific course as well as students’ notetaking practices.

## Results

### Dental literacy in the curriculum for the study programme

Table 3 is based on the SPD and thus represents the intended curriculum [41, 44] in compulsory courses, which means that principles for the programme are given. For Table 3 we have chosen only the courses that specifically address academic or professional literacy in their objectives.

Based on the SPD the intended curriculum include professional literacy skills such as; **a)** oral and written communication with patients in different ages; **b)** oral and written communication with other medical professions; **c)** writing patient records; **d)** judging the patient specific needs (a prerequisite for all following points); **e)** writing referrals; **f)** writing prescriptions, **g)** writing treatment−/revision-plans; **h)** writing clinic specific text such as career-planning, clinical structure, budget, delegations etc.; **i)** reading and interpreting the professional meaning of laws and regulations; **j)** reading and interpreting ethical rules and considerations with the patient in focus; **k)** reading and interpreting patient documentation (records, plans, referrals etc.); **l)** reading and interpreting clinic specific text. For the oral communication the students are expected to use the specific language taught at the different courses during the SPD. However, they are also expected to be able to alter their language in relation to different groups of patients, different professional collaborators as well as the society.

Further, the SPD include academic literacy skills such as; **1)** strategies for database searches; **2)** reading scientific articles, including analysis of their structure, and critical review of their quality; **3)** reading and interpreting ethical rules and considerations for research; **4)** writing, discussing, justifying their and their peer’s study design and methods; **5)** writing their degree project in odontology; **6)** orally presenting and defending their degree project in odontology; **7)** critically review their peer’s degree project in odontology both orally and in writing. For the academic literacy they are expected to use the specific language taught during the course “Degree Project in Odontology”, but also a popular scientific language for the oral presentation of their project, i.e. to be able to adjust the language based on the context..

### Dental literacy in the syllabuses for Orofacial pain and jaw function 1 and 2

The learning outcomes of the module *OPJ 1* are that the student shall: 1) Have good knowledge of the jaw system’s anatomy and function, the dental occlusion and the trigeminal system; 2) Have comprehensive knowledge of temporomandibular dysfunction (TMD), i.e. the conditions that can affect the jaw system; 3) Be able to perform a simplified clinical examination of the jaw system and its function; 4) Independently be able to take alginate impressions from the upper and lower jaw and perform bite registration in retruded contact position; 5) Be able to identify patients with orofacial pain and jaw disorders in a general dental care environment. The learning outcomes of the module *OPJ 2* are that the student shall: 1) Show good knowledge of the clinical examination of the jaw system and its function; 2) Show good knowledge of treatment with occlusal appliances and physical therapies such as jaw exercises; 3) Independently be able to perform a simplified clinical examination of the jaw system and its function; 4) Under supervision, adjust and deliver an occlusal appliance and check its function; 5) Identify and handle patients with orofacial pain and jaw disorders in general dental care environment.

When it comes to *OPJ 1* four of the five learning outcomes point at literacy-related professional knowing, that is terminology related to anatomy, physiology and pathology of the jaw system, i.e., the temporomandibular region, that will be used for communication with colleagues and other related professions, teachers, patients and between students during clinical situations. So, not only knowledge of terminology is sufficient but also communication related to the terminology, with expectations of becoming able to alter the terminology when communicating with e.g. colleagues as compared to patients. All literacy events in this module are based on reading (PowerPoints, handouts and course literature) and writing notes during lectures.

For *OPJ 2*, all learning outcomes point at literacy-related professional knowing. Complementary to the literacy events above, there is both a written instruction (a PowerPoint) at the online-accessible web-platform (Canvas; Instructure Inc., Salt Lake City, USA) as well as a possibility to use the handouts from the lectures that guides students step-by-step for how to adjust and deliver an occlusal appliance, how to do an occlusal analysis (interpret findings), a clinical TMD-examination as well as a follow-up of treatment with the delivered occlusal appliance. Furthermore, there is a video-clip available on their web-platform which demonstrates how to perform the TMD-examination. Finally, there is a physical demonstration for groups of six to eight students each of specific steps related to how to ow to perform the occlusal analysis, the TMD-examination as well as the adjustment and delivery of the occlusal appliance – some (but not all) students take notes during this demonstration. These two modules end with an online and on-site multiple-choice test consisting of five questions.

### Dental literacy as established in teaching practices

All lectures followed a similar pattern: most slides were presented shortly, approximately 1 minute, sometimes even shorter. The teachers defined concepts and explained them based on characteristics of a normal (healthy) anatomy and physiology as opposed to deviances from this.

In general, the slides can be divided into three main types: **a)** *academic content,* that is information found in the course literature that student also can read and which is the basis for examination; **b)** *integrated content,* including information from the course literature that is related to the future profession through for instance complementary illustrations, or real patient cases; and **c)** *professional content,* that is practical application of the core-content.

A recurrent example of *academic content* was basic information about anatomy, physiology and pathology of the temporomandibular region. However, when it comes to *professional content* the most recurrent content was the importance of the patient communication, including the difficulty and necessity of relevant content from the patient history taking (anamnesis). But also, the difficulty in adapting the communication to authorities and other professions. The *integrated content* was found both in the PowerPoint-slides, but also by the questions asked by the students that induced a complementary explanation. Here the teacher used either the white-board (with additional text or illustrations) or patient cases to illustrate how the academic content could be translated to the future profession.

### Students’ dental literacy practices

Within this course two different literacy practices were identified, the first was students note-taking and the second was responses to tests. While the students individually choose the type of note-taking, the student-responses in the written tests are based on the construction of the test and the posed test questions.

#### Students’ notes

The overall picture we identified was that they either 1) copied text from teachers’ PowerPoint-slides, 2) re-formulated text in teachers’ PowerPoint-slides, or 3) wrote complementing text (Table 4).

As Table 4 illustrates none of the students used an exclusive profile type of notetaking. Patterns discerned for students separately is that student 1 had a majority of complementary notes, and a minor amount of copied and reformulated notes. Students 2, 5, 6, 7 and 8 demonstrated similar textual patterns in terms of a majority of notes with complementary character. These students had a few notes with reformulated text. Student 3 had both handwritten notes and digital notes. The handwritten notes were of copied character, while the digital notes were of complementary character. Re-formulations occurred in both handwritten as in digital notes but were few and related to copied text. Students 4 and 9 had similar amount of copied and complementary text, with a minor share of reformulated text. Student 10 had an equal amount of copied, reformulated and complementary text.

General patterns observed for the entire student group, in terms of handwritten notes was copied text from the teacher-constructed PowerPoint, and in terms of digital notes was complementary information. When the slides comprise figures or pictures, students tend to describe and explain them using own words. Less amount of text on slides may enable students to produce independent notes.

Complementary text that comprised teachers’ secondary experience, was discerned as main character of text in student notes. This may indicate that teachers’ secondary experience is an important informal complement in teaching. Same type of complementary text was recurrent in student notes and comprises medical/professional concepts within the specific subject matter. Concepts that students have encountered previously in training, are noted but in relation to the current subject matter. One example is the standardized clinical examination Diagnostic Criteria for Temporomandibular Disorders (DC/TMD) [47], where a specific palpatory pressure of either 0.5 or 1.0 kg depending on structure, is warranted. Other examples were the assessment of degree of abrasion, and the normal range for mouth opening as well as laterotrusive and protrusive movements. This may indicate that students attempt to contextualize concepts to specific areas within the current subject matter.

#### Multiple-choice test

This test was constructed based on one question from each of the five core content areas.

For each core content four to seven questions were constructed, in total 28 questions in the database. The questions in the database are grouped according to subject and level of difficulty. One question from each group was randomly selected by the e-examination program (Canvas, Instructure, Salt Lake City, USA). Thereby, the students did not necessarily answer neither the same questions nor in the same order.

**Fig. 2:**
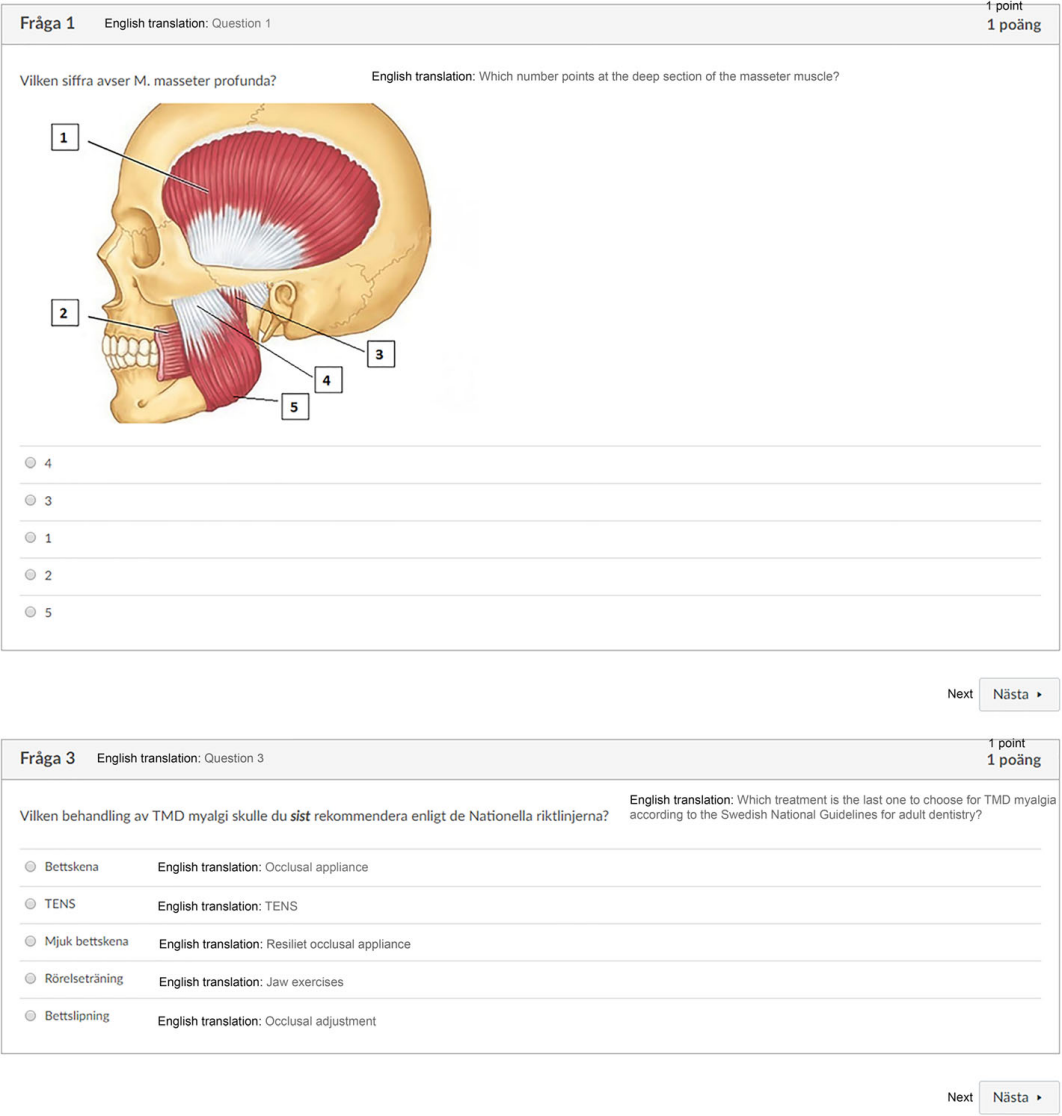
Example of multiple-choice test items. The first question combines an anatomical illustration with numbers in order to identify specific anatomical structures, while the second is a text-based question with multiple answer alternatives

For each question there were from one to three correct answers. Some of the questions were text-based only (question and alternative answers), whereas others combined illustrations and text and/or numbers to identify specific anatomical structures (Fig. 2). Some questions demanded students to judge for instance good or bad choices of treatment according to national guidelines.

For a pass, the students had to achieve 100% correct answers, but had unlimited attempts to perform the test. When analyzing the student performance, i.e., number of attempts to pass, the median (IQR; 25th percentile - 75th percentile) number of attempts was 13 (6–20). Two students passed after one attempt; one student needed 43 attempts.

## Discussion

In the following discussion two themes will be highlighted, firstly regarding literacy practices, and secondly the purposes of literacy practices in dental education.

In the module OPJ1, the majority of learning outcomes (4/5) focused on professional literacy. This involved dental terminology and communication, realized through literacy events of reading and writing. In the module OPJ2, focus was also on professional literacy directed towards the occlusal appliance. Students were engaged in literacy events of reading, watching videos, writing, and following instructions.

Recurrent academic content comprised anatomy, physiology and pathology, while professional content comprised of patient communication and anamnesis. Integrated content was initiated in the teacher-constructed PowerPoint and by student questions.

Teacher provided texts (PowerPoints) summarized main content in the textbook. These slides were uploaded on the web-platform for students in advance, so they had the possibility to access the content before the lecture. A dominant pattern for how teachers used the slides – each slide shortly presented to the students – was that the slides used were multimodal in character. The multimodality was text combined with either illustrative schematic pictures, or movies, or flow-charts of casualties or treatments. There were few exceptions to this pattern, but we identified two types of exceptions:

- the teacher referred to his/her clinical experiences in order to illustrate malfunctions, how to retrieve relevant and correct information from the patient history taking, and how to inform and instruct patients
- students asked questions related to specific content in the PowerPoints and the respective teacher clarified the meaning

For the first exception, we refer to Vygotsky’s [48] concept secondary or social experiences as a resource for newcomers with few experiences. When the teachers shared their experiences with their students, they provided the students with resources for understanding professional (scientific) concepts which mediated professional meaning. Teachers’ (primary) experiences thus became secondary experiences for the students, which according to [48], p. 17) makes it possible for less experienced to “conceptualize something from another person’s narration and description of what he himself has never directly experienced”. The second type of exceptions related to students who shared their lack of understanding, thereby simultaneously making teachers’ clarifying comments or examples available for all students participating in the lecture. These two types of communicative aspects of the literacy practice otherwise dominated by traditional lectures, although with contemporary technology resources.

Notetaking patterns varied between individual students, but general patterns discerned for the entire student group were complementary notes to make available further descriptions of teacher-constructed text, and independent text for describing pictures on the PowerPoint. The complementary text comprised of teachers’ experiences added to teaching, and concepts related to the current subject matter.

We found that so far, the only ones using the textbook were the teachers, who in a way decreased the amount of text the students had to read into the PowerPoint-slides.^1^ Characteristic for the multiple-choice test for the two modules in focus of this article was that the test items demanded reading combined with illustrations. Requirements for a pass grade included 100% correct answers, and median number of attempts for all students were 13.

**Fig. 3:**
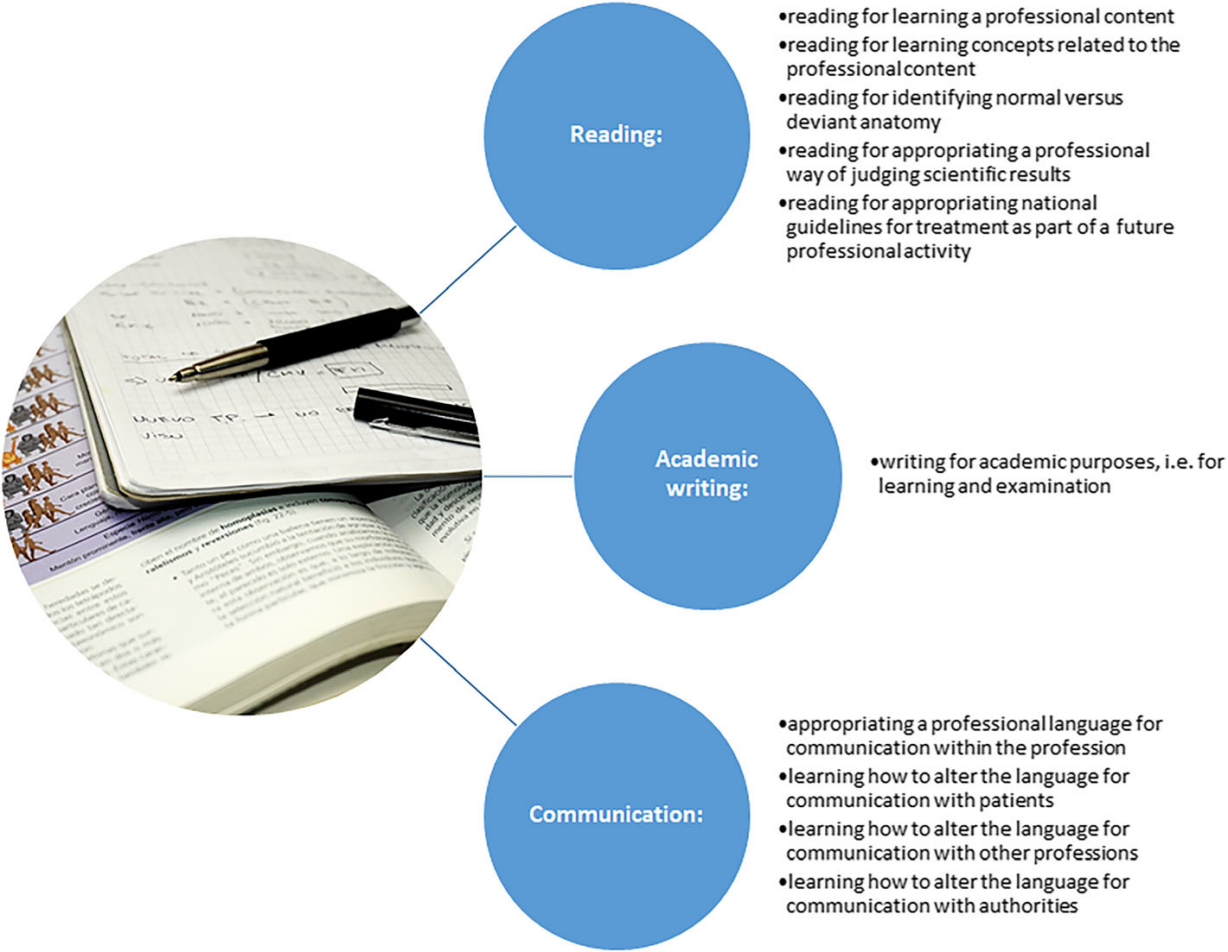
Literacy purposes – an overview of results. This chart divides and exemplifies the literacy practices for reading, academic writing and communication

The purposes identified in the present study regarding literacy practices can be divided in groups of reading purposes, academic writing purposes, and communication purposes. These identified literacy practices have also been shown to be of importance in previous studies in various disciplines such as natural sciences, history, and national economy [12, 14, 37]. Further, just as previous research indicates the importance of progression regarding literacy practices in terms of complexity, and diversity of use [18, 19] this study confirms these findings based on the literacy purposes that emerged in the analysis and elaborated in Fig. 3.

There are just a few studies within the field of dentistry that have addressed different aspects of literacy, but in our knowledge no study has yet addressed literacy in dental education. Results of importance related to the findings reported here specifically concerned three studies. The first one highlighted the student perspective of the impact of communication technologies in their professional communication with patients [49] indicating the importance of our findings to appropriate a professional language for communication within the profession, as well as learning how to alter the language for communication with patients, with other professions, and authorities. Another study [26] showed a high willingness of dental students to adopt and use digital literacy practices, such as smartphones. This, together with the results from a study showing the need of information literacy competences in the era of rapid expansion of the scientific knowledge base [24] highlight literacy practices that also were found in our study, namely usage of digital platforms and computers for reading for learning a professional content and simultaneously concepts related to this content, for identifying normal versus deviant anatomy, and for appropriating a professional way of judging scientific results or national guidelines for treatment as part of a future professional activity.

### Quality of the results

The quality of a case study must be discussed based on the premises of a research paradigm used, in this case the umbrella of qualitative types of data [31]. Here, we emphasize three aspects: **1)** the basic aspect of quality in qualitative research is that researchers give account of their theory of interpretation, in this case the NLS [6, 12, 36, 38], that forms the basis for our understanding and interpretation of data [50]; **2)** the claims for generalizability in qualitative research need to be based on the focus on the empirical basis a posteriori, rather than on the kind of a priori probability theoretical assumptions that form the basis for descriptive statistics [51]. Furthermore, in 2009 Nordenstam [52] emphasized firstly the accuracy and power of single cases as examples of something specific that can be used as a basis for comparative generalization, and secondly the importance of concepts for dissemination of the example in the pursuit of generalization; **3)** validity is a concept with many meanings, in this study we follow two of the aspects discussed by Kvale in 1995 [53] (see also Cohen & Manion [31]): firstly, the craftmanship of the researchers throughout the research process requires careful design, constant questioning of the process on the one hand, and made transparent to others on the other. In this article, the findings related to students’ notes are based on patterns of similarity between the cases. As the analytical process builds on interpretation of data in relation to a specific theoretical basis, the identified patterns must both reduce data into meaningful patterns and still not distort the complexity in data [51]. For validation of the results of this case, we turn to relevant research communities: NLS-studies on higher education, based on similarities in context: the interest in professional programs supports our results as valid for *this type of* education. However, none of these studies has so far examined dental education, therefore the results are so far but one of the pieces in a bigger jigsaw puzzle: the concept itself, *literacy practices*, assumes that these practices are situated and related to specific purposes [5, 6, 8]. The overarching purposes of dental education are twofold: to prepare for the profession, dentistry, and to prepare for future research within dentistry. Furthermore, the communicative validity – e.g. when the results are made available for specific communities for a dialogue, when the example is made available for validation by others in the research communities of dental/medical scholars.

## Conclusion

In conclusion, the present study reveal that teachers’ literacy practices rely on lectures, based on PowerPoints, as well as the multiple-choice tests, represent literacy practice common within disciplines like science in academia, as is the multimodality of the content in these PowerPoints: a combination of text and pictures. Part of the PowerPoints relate to the basis for professional knowledge – students’ learning (anatomy and physiology) – whereas others relate to professional use of specific texts. Characteristic for students’ literacy practices is that they either copy text from teachers’ PowerPoint-slides, re-formulate text from teachers’ PowerPoint-slides, or write complementing text to teachers’ PowerPoint-slides. Further, the students individually choose type of note-taking based on situation. In the two modules – of the dental education analyzed – the literacy practices provide both a knowledge base and a language for a basis for *reading*, *writing*, and *communication* purposes. The study also show that academic and professional literacy are closely connected through recurrent integration.

## Data Availability

Nikolaos Christidis can provide the data and material upon request.

## Footnotes

1 However, these two modules are only part of the course and will be followed by OPJ 3 and OPJ 4 in semesters 8 and 9. During the 8th semester three larger multiple-choice examinations will take part. One will test anatomy, one diagnostics according to DC/TMD, and one final examination of the entire course content. At the end of semester 9 a written assignment will be handed in followed by an oral examination on the present assignment.
